# Perineal Section

**Published:** 1875-05-01

**Authors:** E. R. Willard

**Affiliations:** Wilmington, Ill.


THE
### MEDICAL EXAMINER.
A
Semi-Monthly Journal of Medical Sciences.

EDITED BY N. S. DAVIS, M.D., AND F. H. DAVIS, M.D.

No. IX. CHICAGO, MAY 1, 1875. VOL. XVI.

### Original Communications.

### PERINEAL SECTION.

By E. R. WILLARD, M.D., WILMINGTON, ILL.

*Extract from an article read before the Will County Medical Society, April 6th, 1875.*

THERE has been so much written the past ten years upon external perineal section, or urethrotomy, that it would seem a correct appreciation of the value of the operation had already become fully established by the profession.

Although such may seem to be the fact from the published accounts of cases, still, when we come to look for these cases, we find them confined to a small, select number of surgeons, and those mostly of a number who make a specialty of that department of surgery.

So far back as the seventeenth century, such men as Desault and Chopart condemned the operation on account of the great mortality attending it, and thus brought it into disrepute. In 1817 Dr. A. H. Stephens performed the operation with entire success, followed soon after by Jameson, Rodgers, and Warren, all of this country, and with the same uniform good result.

Although this was the case fifty years ago, and all well-informed surgeons of a later date must have been fully conversant with these facts, still the operation has seldom been performed since that time unless by specialists.

Prof. Van Buren says it is "one of the most difficult and uncertain proceedings of surgery."

Prof. Markae says "the dangers of the operation itself can hardly be separated from those of the disease for which it is performed," while Prof. Gross observes that "the operation is by no means free from danger, and requires the most consummate skill for its successful execution."

In looking over some notes taken in 1851, from clinical lectures of

Prof. Brainard, I find that he regarded the operation as more hazardous to the patient than that of puncturing the bladder above the pubis, or through the rectum, as instanced more particularly by a case operated upon by the former method (puncture above the pubis) by Prof. Brainard at that time. The case, a man over sixty years of age, had retention of urine thirty-six to forty-eight hours. A trocar was thrust in above the pubis, and the water drawn off. Result: the man died in a few days.

Prof. Hamilton states in his late work that "the operation will require more skill and patience than almost any other in surgery." With such authority it is no wonder the operation is confined mostly to specialists, and that most of the well-informed physicians and surgeons throughout the country are more inclined to puncture above the pubis, or through the rectum, than perform urethrotomy.

The main consideration in the performance of this operation is the danger attending it; and, so far as we are able to judge from the writings of such men as Syme, Miller, Thompson, Smith, and Gouley, the operation is not any more dangerous than that for inguinal hernia; that is, the mortality attending the operation proper only being about six to seven per cent.

Taking into consideration the above statement, together with the fact that the operation will afford permanent relief, with the certainty of ultimate cure, provided a sound is used occasionally for some time thereafter, in almost all cases treated by this plan it would seem that any other operation would not be thought of for a moment, unless the prospects for success were the same.

Before giving the following case, I would state that Prof. Gouley is entirely opposed to tying in a catheter after the operation, on account of, first, its uselessness, and second, its occasional danger. First, because the urine does not pass out through the catheter, but dribbles down by its side. Second, the irritation caused by the catheter is ten-fold more than that caused by the urine, provided the retention of the catheter would prevent the flow through the wounds.

Case ——. July 21st, 1874, I was sent for by Dr. Watson to see Mr. William Gardner, a coal miner at the B. shaft, some five miles distant. He was suffering from retention of urine, caused by stricture of the urethra, consequent upon gonorrhœa, for which he had been treated by a number of physicians at different times the past three years, none of whom succeeded in passing a catheter.

As the retention was only partial, and the bladder never distended but slightly above its natural capacity, after which a capillary stream could be voided sufficient to relieve the urgent symptoms. He had continued in this uncomfortable condition for the past three years, up to a week previous to my seeing him, at which time he became very much worse, in so much that no urine had passed the urethra for seventy-two hours. The bladder was distended to its utmost capacity, reaching above the level of the umbilicus, giving the appearance of a woman between the sixth and seventh months of pregnancy, the distention having continued so long and to such an extent that the nervous sensibility had become partially destroyed, in so much that he expressed himself as quite comfortable as compared with the two or three days' previous suffering. I tried to introduce catheters and sounds of various sizes, all to no purpose. I then called in Drs. Merriman, Le Caron, and Willard to assist in the operation of perineal section.

Dr. Merriman having had considerable experience in passing the capillary probe-pointed whalebone bougies, as recommended by Prof. Gouley, thought he could enter the bladder without difficulty with one of these guides. After some two hours' unsuccessful attempts by Drs. Merriman, Le Caron, and myself, to make one of these guides pass the obstruction and enter the bladder, by introducing one after another, having as many as six and seven caught in the same place at one time, we were about giving up, when I succeeded in slipping one of these guides into the bladder.

We then withdrew the other guides, and took a small-sized catheter, perforated the end, and slipped it over the remaining guide down to the stricture, with the intention of forcing it through into the bladder; but this we found to be impossible, as all the pressure the catheter was able to sustain was not sufficient to overcome the obstruction. Finding further efforts in that direction of no avail, we removed the perforated catheter from off the guide and passed a silver catheter down to the point of stricture, put the patient in the position for lithotomy, with Dr. G. E. Willard in charge of the ether, Dr. Merriman the catheter, and Drs. Le Caron and Watson to assist as occasion might require.

I made an incision one inch and a half in length in the median line of the perineum, dividing all the tissues down to the catheter, opening the urethra a little anterior to its distal end. I then introduced a small grooved directory through the perineal wound into the urethra, pressed it firmly against the stricture, placed the point of the knife in the groove, and divided the stricture freely, so that Dr. Merriman was enabled to pass the full-sized catheter into the bladder readily. The bleeding was very light, and entirely checked by sponging with cold water. The catheter was tied in and left *in situ* for forty-eight hours. Although the catheter was retained in the bladder the first two days after the operation, still the urine passed entirely by the perineal wound at its side, thus showing that the retention of the catheter was of no use. The only reasons ever alleged for retaining a catheter *in situ* for any length of time after the operation, is to prevent the urine from coming in contact with the recently cut surfaces, thereby preventing the rapid granulation and union of the wounded tissues.

In two or three days after withdrawing the catheter the urine began to pass the urethra, and in a week none came through the perineal wound, granulation being so rapid that in four weeks the wound was entirely healed. In short, the patient made an excellent and rapid recovery.

